# Eggplant Flour Addition in Cookie: Nutritional Enrichment Alternative for Children

**DOI:** 10.3390/foods11121667

**Published:** 2022-06-07

**Authors:** Jaqueline Machado Soares, Flávia Teixeira, Mayra Lopes de Oliveira, Luane Aparecida do Amaral, Tainá da Silva Fleming de Almeida, Gabriel Henrique Oliveira de Souza, Lais Maluf Hokama, Bruna Menegassi, Elisvânia Freitas dos Santos, Daiana Novello

**Affiliations:** 1 Department of Nutrition, Health Sciences Sector, State University of Midwest, Guarapuava 85040-167, Brazil; nutrijaquesoares@gmail.com (J.M.S.); teixeiraflavia19@gmail.com (F.T.); mayra.lopes2010@gmail.com (M.L.d.O.); luapamaral@hotmail.com (L.A.d.A.); 2 Faculty of Pharmaceutical Sciences, Food and Nutrition, Federal University of Mato Grosso do Sul, Campo Grande 79070-900, Brazil; tainaafleming@gmail.com (T.d.S.F.d.A.); g.henriqueoliveirasouza@gmail.com (G.H.O.d.S.); maluf.lais@gmail.com (L.M.H.); elisvania@gmail.com (E.F.d.S.); 3 Faculty of Health Sciences, Federal University of Grande Dourados, Dourados 79825-070, Brazil; brunamenegassi.nut@gmail.com

**Keywords:** product development, eggplant flour cookie, children, physicochemical properties, sensory properties

## Abstract

This research aimed to evaluate the effect of adding different levels of eggplant flour in cookie on the physicochemical and nutritional characteristics and to verify the sensory acceptability among children. Four eggplant flour cookie formulations were prepared: EF0 (or standard), EF2.5, EF5.0, and EF7.5 (Eggplant Flour 0, 2.5, 5.0 and 7.5%, respectively). The sensory acceptability, physicochemical and nutritional composition were evaluated. The eggplant flour addition of 7.5% to cookie reduced the acceptability (*p* > 0.05). The samples EF5.0 and EF7.5 showed higher diameter, expansion and thermal factor, while the EF0 and EF2.5 had higher thickness (*p* < 0.05). The flour addition significantly increased the hardness, Water activity (Aw), Titratable Acidity (TA) and Soluble Solids (SS) in the cookie, however, L* a* and b*, pH and SS/TA ratio values were reduced (*p* < 0.05). Increased levels of ash, dietary fibers, ascorbic acid, anthocyanins, total phenolic compounds and antioxidant activity were verified on the cookie after eggplant flour addition. Meanwhile, there was a reduction in energy and carbohydrate values. It is concluded that eggplant flour addition up to 5% in cookie maintains the sensory acceptability similar to the standard product when evaluated by children. In addition, it can be considered a viable alternative to improve most of the physicochemical and nutritional characteristics of the product.

## 1. Introduction

The eggplant, botanically classified as *Solanum melongena* L., is a non-tuberous species of great economic and agronomic importance for Solanaceae family [1,2]. Its cultivation has begun more than 4000 years ago at Southeast Asia [2] and, currently, is spread all over the world, with an annual production of 50 million tons approximately [3]. In Brazil, there are more than 11,000 farming establishment eggplant producers. On average, 70,000 tons are cultivated by year with a special focus for the Southeast and South region of the country, which holds the biggest production [4].

Although different varieties of eggplants exist, the most commonly cultivated has an oblong format, shining deep-purple color and green stem. The vegetable has characteristics of berries and it contains many edible soft seeds [2,5]. Its composition presents high levels of vitamin A, folate, vitamin k, vitamin C, potassium, phosphorus, magnesium and calcium [6]. Besides, the eggplant has antioxidant compounds such as phenolic compounds and anthocyanin. The phenolic compounds decrease intestinal absorption of monosaccharides, which helps to regulate the circulating glucose levels in patients with diabetes mellitus. The anthocyanins, on the other hand, help with hyperlipidemia treatment and in atherogenic cardiovascular disease prevention by inhibition of lipid peroxidation. Eggplants also present a low caloric level, which could be used in weight reduction diets [2,5,7,8].

The per capita consumption of eggplant is small and it is 0.88 lb/yea [9]. The low acceptability is due to the presence of the nicotinoid alkaloids which confers a bitter taste to the vegetable [2]. In childhood, particularly, these compounds are noticed more intensely, once that children are more sensitive to the bitter taste when compared to adults [10]. School-age children usually present low acceptability by vegetables, about 78% of them dislike eggplant [11]. Besides, the general consumption of vegetables among kids is approximately 150 g/day [12], lower than the World Health Organization (WHO) recommendation, which recommends 400 g for fruits and vegetables [13].

Some technological alternatives have been used to introduce the eggplant into the usual diet, as an example the use of eggplant flour [8] in cookies [5], breads [14], cakes [7] and pasta in general [15]. Its flour can be obtained by a process of lyophilization, which consists of removing water from the food by sublimation at a high vacuum, however, it has high-level production costs. Thus, the vegetable dehydration in oven drying or conventional oven seems to be more accessible strategy. Furthermore, when compared to lyophilization, drying may increases the antioxidant capacity, retains more phenolic compounds [16,17], flavonoids and anthocyanins [16] in eggplants.

Panification products such as biscuits, bread, cakes and pasta products are widely accepted by the world population. In Brazil, biscuits represent the most profitable category for the market, with a per capita consumption of 7.2 kg/year. Currently, there are several options being sold. Biscuit- type cookies stand out due to the constant increase in production and sales. It is estimated that 29,000 tons were produced in 2019, with sales of R$ 967,000 [18]. Cookies are often consumed by all age ranges due to the practice, storage ease, long shelf-life [19]. In the specific case of children, cookies stand out due to their attractive attributes, great appearance, crunchy texture, sweetened taste and flavor [20]. Nevertheless, they can contain high lipids, sugar levels, and low fiber, vitamins, mineral levels, which is not recommended for child consume. In that regard, the addition of fruits and vegetable flour can be a better strategy to improve the nutritional value of cookies [5,7,8,21]. However, there are acceptable levels of these flour addition in food products, as high concentrations can cause sensory and technological prejudice. The addition of 10% eggplant flour in cookie reduced the acceptability for taste and appearance attributes among adults [8], raised the hardness and promoted color darkness in the product product [5]. In this context, this research aimed to evaluate the effect of adding different levels of eggplant flour in cookie on the physicochemical and nutritional characteristics and to verify the sensory acceptability among children.

## 2. Materials and Methods

### 2.1. Ethical Approval

The study was conducted in accordance with the Declaration of Helsinki and the ethical approval was conceded by the Ethics Committee of Midwest State University, protocol number 3,089,447/2018. This research included children aged 7–10, enrolled between 2nd and 5th grade in 10 public schools in the urban area of Guarapuava, PR, Brazil. All children declared their consent and had informed consent from the legal guardian to participate in the research.

### 2.2. Food Acceptance Evaluation

In order to evaluate the food acceptance a questionnaire containing food designs belonging all food groups was elaborated: cereals; fruits; vegetables; dairy; meats and eggs; beans and oilseeds; oils and fats; sugar and sweets [22]. The foods included in this questionnaire were marketed in region of Guarapuava, at low cost and accessible to children, besides being usually offered in school meals. A total of 214 children received and responded to printed instrument with “x” whether they liked the described food or not. The purpose of applying this instrument was to identify the healthier and most nutritious food with lower acceptance among children and then use it as an ingredient in the elaboration of a new product. The food with lower acceptance was eggplant, being used as ingredient in the cookie elaboration.

### 2.3. Eggplant Flour Elaboration

The eggplants (*Solanum melongena* L.), with better visual appearance, smooth surface without imperfections and bright deep-purple color, were purchased (20 kg) from a local market in Guarapuava. The fruits were washed in potable running water and sanitized in sodium hypochlorite solution (200 ppm) for 15 min and rinsed under running water again [23]. Then were integrally sliced (approx. thickness of 5 mm) and dried in oven drying with air circulation (Pardal^®^, SEDi-C 40L model, Rio de Janeiro, RJ, Brazil) at 65 °C for 24 h. After they remained at room temperature (22 °C) up to total cooling, they were milled in a mill (Tecnal^®^, TE-631/4 model, Piracicaba, SP, Brazil) and passed through a sieve with 32 mesh/Tyler opening (Bertel^®^, 5 mm, Caieiras, SP, Brazil), obtaining a final yield flour of 1.5 kg.

### 2.4. Cookie Formulations

**Figure 1:**
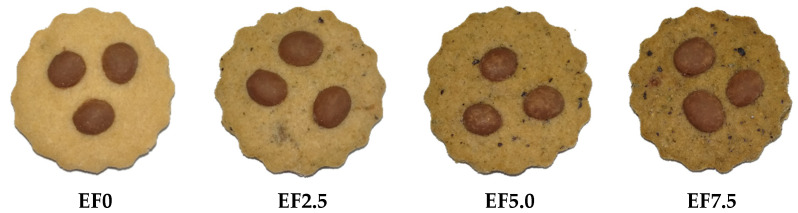
Cookie formulation added to different levels of eggplant flour.

Four eggplant flour cookie formulations were prepared: EF0 (or standard), EF2.5, EF5.0 and EF7.5 (Eggplant Flour 0, 2.5, 5.0 and 7.5%, respectively) (Figure 1). These percentages were defined through preliminary sensory tests performed with the product (data not shown). The following ingredients were also used: wheat flour (F1: 48.0%, F2: 45.5%, F3: 43.0%, F4: 40.5%), eggs (16.6%), butter (13.8%), chocolate drops (11.0%), brown sugar (9.7%), vanilla essence (0.5%) and chemical yeast (0.4%). The other ingredients, besides eggplants, were also purchased from local markets in Guarapuava.

For the cookies’ elaboration, the butter, brown sugar and vanilla essence were mixed using a domestic blender (Arno^®^, SX34 model, Itaqui, SP, Brazil). Then, the eggs were added and mixed until homogenization. Afterwards, the wheat and eggplant flour were incorporated to dough. The chemical yeast and chocolate drops were added and mixed manually at the end of preparing. The cookies were molded in circular format (approx. 4 cm of diameter) and baked at 180 °C for 12 min in electric oven (Fischer^®^, hot grill model, Brusque, SC, Brazil) preheated. After that, they rested until reach room temperature (22 °C) and were packed in hermetically sealed plastic containers until the analysis.

### 2.5. Sensory Evaluation

The sensory evaluation was performed in available classrooms at the schools with the same children who answered the questionnaire described in Section 2.2. Each test was carried in portable sensory booths individually and each child was instructed by the researchers to fill the answers. The appearance, flavor, taste, texture and color were evaluated with a 7-point structured facial hedonic scale, going from 1 (Super bad) to 7 (Super good), adapted from Kroll [24]. Furthermore, acceptance and purchase intention questions were applied with a 5-point structured facial scale (going from 1—Dislike a lot/Would not buy it to 5—Like a lot/would certainly buy it). The consumers received 10 g of each sample on white disposable plates (15 cm) coded with three-digit numbers, in a randomized and balanced way. A glass of water was provided for cleaning the palate. The formulations were offered in a monadic sequence. The Acceptability Index (AI) was calculated according to the formula: AI (%) = A × 100/B (A = mean grade obtained for the product; B = maximum grade given to the product) [25].

### 2.6. Physicochemical and Nutritional Analysis

To the physical evaluation cookies from the same batch (*n* = 10) were chosen randomly. The mass parameters, diameter and thickness were determined according to the precepts describe by the American Association of Cereal Chemists [26]. Diameter and thickness were measured using an analogical pachymeter (Mitutoyo^®^, 530-104BR model, Kawasaki, OL, Japan). The expansion factor was obtained by the ratio diameter/thickness of the cookie. The thermal factor was obtained by the ratio between the pre-bake and after baking mass values. Each unit of cookie was considered as an experimental replay.

The hardness was measured with five replicates in all formulations within 24 h after baking. A texture Analyzer with a probe HDP/BSK (a knife simulator) was used (Stable Micro Systems^®^, TA.XT Plus C model, Godalming, SY, UK), interconnected with the Exponent Lite Software (Stable Micro Systems^®^, version 4.0.8.0, Godalming, SY, UK) installed in a computer. The cookie (approx. 1 cm of thickness and 4 cm of diameter) was compressed up to break, the strength spent was defined as cutting force. The measurement conditions were maintained as the pre-test speed at 1 mm/s, test speed at 3 mm/s, post-test speed at 10 mm/s and trigger force of 0,1 N.

The color was analyzed in five replicates for eggplant flour and cookies. Chromatic parameters were obtained in a colorimeter (Konica Minolta^®^, Chroma Meter CR 4400 model, Tokyo, HSJ, Japan) using CIELAB (L*, a*, b*) color systems according to Commission Internationale de l’Écleirage. L* defines Lightness (0 = black, 100 = white), a* indicates red (positive a*) or green value (negative a*) and b* indicates yellow (positive b*) or blue value (negative b*) [27].

The chemical determinations were performed in triplicate on the eggplant flour and all the cookie formulations. The water activity (Aw) was determined by an Aw analyzer (Novasina^®^, Labswift model, Lachen, SZ, Switzerland) at 22 °C; pH, measured by a bench pH meter (Tecnopon^®^, mPA-210 model, Piracicaba, SP, Brazil), calibrated with pH 4.0 and 7.0 buffers; Titratable Acidity (TA) evaluated according to the Association of Official Analytical Chemistry (AOAC) [28]. Initially 5 g of sample was added to 95 mL of distilled water. The titration was made with the aid of digital burette (Brand^®^, Z567132 model, Essex, CT, USA), containing sodium hydroxide solution (0.1 mol L^−1^) until reaching pH 8.1. The results expressed as g of citric acid 100 g^−1^; Soluble Solids (SS) were measured according to AOAC [28] with solubilization of the samples in a known volume of distilled water (1:3, *m/v*) at room temperature (22 °C). Two to three drops of the sample filtrate were used and the reading was performed on a bench-top refractometer (Bel^®^, RTA-100 model, Monza, MB, Italy). Values were expressed by °Brix (scale from 0 up to 95 °Brix–minimal score of 0.25 °Brix); SS/TA ratio which was obtained by dividing SS and TA values.

For the nutritional composition analysis, the samples were evaluated in triplicate according to the following measurements: moisture determined in a drying oven at 105 °C until weight constant (g 100 g^−1^); ash (g 100 g^−1^) analyzed in a muffle furnace at a temperature of 550 °C; protein (g 100 g^−1^) analyzed through to the Kjeldahl method. The factor 6.25 was used for the nitrogen conversion into crude protein [28]; lipid (g 100 g^−1^) by the hot extraction method with Soxhlet extractor and petroleum ether [29]; carbohydrate (g 100 g^−1^) by difference method (% carbohydrate = 100 − (% moisture + % ash + % protein + % lipid + % dietary fiber); total, soluble and insoluble dietary fiber were determined according to the AOAC 991.43 method. Total fiber and insoluble dietary fiber by enzymatic method and the soluble dietary fiber were calculated by the difference of the total and insoluble dietary fiber results [28]; energy value (kcal 100 g^−1^), calculated by the values recommended for lipid (9 kcal g^−1^), protein (4 kcal g^−1^), carbohydrate (4 kcal g^−1^) and fiber (2 kcal g^−1^) [30]. The Daily Reference Value (DRV) for fibers was calculated for 30 g of the sample (three cookie units), based on the recommended daily mean intake values for children (7 to 10 years) [31], resulting in 26.8 g day^−1^.

The bioactive compounds and antioxidant activity were evaluated in triplicate in the eggplant flour and cookies.

Ascorbic acid was estimated by the titrimetric method of AOAC (2016) modified by Benassi and Antunes [32]. Samples were homogenized with 1% oxalic acid (1:10 *m*/*v*) and titrated against 2,6-dichlorophenol-indophenol (DCFI) dye. The results were expressed in mg ascorbic acid 100 g^−1^.

The samples submitted to the analysis of anthocyanins, total phenolic compounds and antioxidant activity were extracted using the solvent ethanol. 2.5 g of sample plus 15 mL of 80% ethanol solvent were used. The samples were vortexed (1 min) at room temperature (22 °C) in low light and placed in a water bath at 40 °C for 10 min (in the extraction for analysis of antioxidant activity the water bath was not performed). The homogenate was centrifuged at 5000× *g* rpm for 10 min (22 °C) and the supernatant was recovered. Then the supernatants were pooled and the volume standardized to 25 mL.

The quantification of anthocyanins was made by the differential pH method [33]. Two buffer solutions were used, a KCl buffer (0.025 M–pH 1.0) and a CH3COONa buffer (0.4 M–pH 4.5). For each repetition, two test tubes were used with the following constituents: (a) 0.3 mL of the ethanolic extract was added with 2.7 mL of KCl buffer; (b) 0.3 mL of the ethanolic extract was added with 2.7 mL of CH3COONa buffer. The contents were mixed well and remained at room temperature (22 °C) for 15 min. Then readings were made on a spectrophotometer (Agilent Technologies^®^, Cary 60 UV model, Santa Clara, CA, USA) at 520 and 700 nm. The reading at 700 nm was performed to discount the turbidity of the sample. The results were reported as mg of cyanidin-3-glucoside (C3GE) 100 g^−1^.

The Total Phenolic Compounds (TPC) was determined based on the Folin-Ciocalteau method described by Woisky and Salatino [34], using gallic acid as a standard for the calibration curve. In a test tube, 0.5 mL of the ethanolic extract was added 2.5 mL of the Folin-Ciocalteau reagent (10%). After 5 min, 2 mL of Na_2_CO_3_ (4%) was added. The contents were well mixed and left at room temperature (22 °C) without light for 2 h. Absorbance was measured on a spectrophotometer (Agilent Technologies^®^, Cary 60 UV model, Santa Clara, CA, USA) at 765 nm. The results were expressed as mg of Gallic Acid Equivalent (GAE) 100 g^−1^.

The Antioxidant Activity (AA) was evaluated using the 2,2′-azino-bis (3-ethylbenzothiazoline-6-sulfonic acid (ABTS) radical method, as proposed by Re et al. [35] with modifications. The formation of the ABTS^•+^ radical was given by homogenizing equal parts of ABTS (7 mM) and potassium persulfate (140 mM) with storage for 16 h at room temperature (22 °C) in the dark. The solution was then diluted in phosphate buffered saline (PBS) (5 mM–pH 7.4) until the absorbance of 0.70 nm ± 0.05 nm at 734 nm. This solution was used to determine the standard curve (with Trolox up to 100–2000 μM) and samples. In a test tube, 0.5 mL of the ethanolic extract was added with 0.2 mL of ABTS^•+^ solution. The contents were mixed well and remained at room temperature (22 °C) for 2 min. Spectrophotometer (Agilent Technologies^®^, Cary 60 UV model, Santa Clara, CA, USA) reading was performed, absorbance at 734 nm and the results were expressed as µmol Trolox equivalents 100 g^−1^.

### 2.7. Statistical Analysis

The software R (Lucent Technologies^®^, version 3.5.3, Alpharetta, GA, USA) was used in this study [36]. The data were analyzed for normality (Shapiro–Wilk) and homogeneity of variance (Box–Cox). Then the data were subjected to analysis of variance (ANOVA). The means were compared by Tukey’s test at 5% significance level (*p* < 0.05).

## 3. Results and Discussion

### 3.1. Food Acceptance

The foods that children did not like more often were eggplant (80.8%, *n* = 173), chard (76.2%, *n* = 163), watercress (75.5%, *n* = 162), radish (65.0%, *n* = 139) and chayote (64.0%, *n* = 137). Hanson et al. [37] and Cain et al. [11] found similar results studying the feeding preferences in school-age children in The United States and Brazil, respectively. Low acceptance of vegetables among children is associated to high amount of antioxidant compounds, like phenolics, flavonoids, isoflavones, therpenes, glucosinolate, which are present in these foods and promote a bitter and/or astringent taste [38,39]. Usually, children also do not consider the appearance and texture of vegetables attractive, which may reduce the acceptance [37]. For this reason, the development of studies that encourage the consumption of these foods through different ways, is a good strategy to improve acceptance among children.

### 3.2. Sensory Analysis

In Table 1, the sensory scores of cookies are presented. The 7.5% eggplant flour addition in cookie reduced the acceptance (*p* < 0.05) among children. Similar results were verified by Brasil et al. [40] evaluating the addition of eggplant flour (10%) in bread among adults. The high content of phenolic compounds in the eggplant flour (1540 mg GAE 100 g^−1^) [7] promote characteristic flavor and residual bitter taste [38], which could explain the lower score for the EF7.5 formulation. Besides, the appearance change due to eggplant flour addition could have an impact on the acceptance. Chung et al. [41] explain that appearance of the product of food is considered determining factor for acceptance or rejection among children, because it is the first basis of judgment and affects other sensory perceptions. During the cookie prepare was verified that addition of higher levels of eggplant flour resulted in a darkness color product. This effect is due to the anthocyanins presented in eggplant, which has purplish coloring. Furthermore, eggplant flour addition promoted a higher hardness on the dough, due to its high fiber level and low gluten content [5]. Despite the sensory scores being reduced for the EF7.5 formulation, all the formulations presented AI ≥ 70%, indicating good sensory acceptance [25].

### 3.3. Physicochemical and Nutritional Analysis

The samples EF5.0 and EF7.5 showed higher diameter, expansion and thermal factor, while the EF0 and EF2.5 had higher thickness (*p* > 0.05), as expressed in Table 2. The eggplant flour addition increased the hardness proportionally in all formulations (*p* > 0.05). Cookies prepared with eggplant flour have high fiber content, which increases water absorption and, consequently yield, once it shows a lower shrinkage [5,42]. However, fibers also make the dough more brittle and hard after baking, raising the product hardness [43,44]. Particularly among children, a hardness raise can be a limiting factor for chewing, once that during the school-age occurs the transition from mixed dentition [45]. Furthermore, texture changes can affect other sensory characteristic, as appearance and taste product [41]. Gluten is also a factor that interferes at the physical characteristics of cookies. It is an insoluble protein complex responsible to confer extensibility and elasticity to dough during the mixing process of gliadin and glutenin (present in wheat) with water and mechanic force [46]. So, the substitution of wheat for eggplant flour (gluten-free), reduces thickness of cookie after baked, as shown in other studies [5,47].

The eggplant flour addition reduced L*, a* and b* values in cookie, especially after 7.5% addition. A lower lightness occurred due to wheat flour shows higher values of L* (94.2 ± 1.5) [48] if compared to eggplant flour (67.7 ± 0.92, evaluated by the authors). Besides, enzymatic oxidation of phenolic compounds in the flour causes darkness in the product [2]. Raised levels of anthocyanin in eggplant flour (7.5 mg 100^−1^) [49] also reduce values of red and yellow. For cookies, the ideal Aw level must be under 0.6 [50]. Thus, the Aw raised after eggplant flour addition may reduce crunchiness of cookie. Furthermore, flour of fruits and vegetables, usually present higher Aw level, which may increase perishability and reduce microbiological stability of the product.

The chemical characteristics results of eggplant flour and cookie are described in Table 3. The TA and SS increased proportionally with eggplant flour addition in cookie, while pH and SS/TA ratio decreased (*p* > 0.05). According to the scientific literature the presence of phenolics in the eggplant flour, such as chlorogenic acid (1.73 mg g^−1^), caffeic acid (0.19 mg g^−1^) and ferulic acid (0.04 mg g^−1^) [7] raise the acidity of flour, and consequently of the product. This effect causes a bitter taste to the food [7,38], which may reduce sensory acceptance, as was observed in cookies with higher eggplant flour level (7.5%). On the other hand, a higher SS/TA ratio can indicate a more pleasant taste product to the consumer, as sugar and acids are equilibrating [51].

In Table 4 nutritional composition and antioxidant activity of eggplant flour and cookie formulations added of different levels of eggplant flour is presented. The oven drying method allows for a nutrient concentration in food products as occurred in eggplants flour in this research. Similar results were observed by other researchers [5,7,16,52]. However, small differences are possible as the composition can be influenced by variety of the plant, conditions for cultivation, harvesting and processing inputted [53].

The moisture content of eggplant flour is according to Brazilian legislation (15%) [54], and other countries such as Mexico (15%) [55] and United States (14%) [56]. Moreover, it is within the range normally observed for that product (1.5% to 8.5%) [16]. Addition of eggplant flour in cookies increased moisture levels (*p* > 0.05). According to Uthumporn et al. [5], the eggplant flour incorporation on cookies may increases moisture level due to its high amount of fibers. The hydroxyl groups from fibers are capable of connecting to free water molecules through hydrogen bonding [57] consequently retaining more water.

The ash content observed in the eggplant flour impacted the increased ash level in cookie formulations. It is known that ash reflects the concentration of minerals in its composition. A study by Rodriguez-Jimenez [16] on the mineral content of eggplant flour showed a high concentration of minerals such as potassium (2396.0 mg 100 g^−1^), magnesium (158.1 mg 100 g^−1^), calcium (130.9 mg 100 g^−1^), sodium (68.1 mg 100 g^−1^), iron (2.9 mg 100 g^−1^), manganese (2.5 mg 100 g^−1^), zinc (2.1 mg 100 g^−1^) and copper (1.0 mg 100 g^−1^). During school-age, a higher consumption of these micronutrients can be beneficial, as it is associated to health growing and appropriate physical, cognitive and emotional development of children [58,59].

The protein and lipid amounts in eggplant flour are similar to wheat flour (12.0 g 100 g^−1^ and 1.7 g 100 g^−1^, respectively) [60]. Therefore, there was no alterations for these nutrients in cookie (*p* > 0.05). The opposite was verified in levels of carbohydrate and energy, as eggplant flour presents lower amount when comparing to wheat flour (72.5 g 100 g^−1^ and 361 kcal 100 g^−1^, respectively). With it, there was a reduction in carbohydrate and energy levels in EF5.0 and EF7.5 formulations.

There was an increase on total fiber content to EF2.5 (57.1%), EF5.0 (114.3%) and EF7.5 (171.4%) with regard to EF0, as eggplant flour has a high fiber level (34.5 g 100 g^−1^), corroborating with literature [5,47]. The 5% eggplant formulations may be considered a dietary fiber source product, as it has more than 3% of fiber composition [61]. That way, the consumption of 30 g of cookie or three cookie units with 5% vegetable flour added (acceptance similar to EF0 formulation) achieve 3.4% of the DRV of dietary fibers for children [31].

The eggplant flour addition in cookie increased proportionality the ascorbic acid, anthocyanins, total phenolic compounds and antioxidant activity (*p* < 0.05). A similar effect was related by Uthumporn et al. [5] analyzing cookie with eggplant flour (0, 10 and 15%). Substances such as ascorbic acid, anthocyanins and phenolics are considered phytochemicals by presenting health benefits. These compounds are positively correlated to antioxidant activity [62], which can eliminate free radicals due to their chemical structure [16]. Gürbüz et al. [2] showed that the eggplant bioactive compounds have anti-inflammatory, antiangiogenic, anti-obesity, anti-diabetic, anti-carcinogenic and antiviral properties. That demonstrate the viability of using eggplant flour to elaborate food products with better nutritional profile, especially for children, which increasingly consume low nutritional level food.

## 4. Conclusions

The eggplant flour is a viable alternative as cookie ingredient. The 5% level addition of this flour in product is well accepted by children at school-age, obtaining similar acceptability to the standard product and good commercialization expectation. However, eggplant flour harms some of the physicochemical characteristics in the cookie, such as water activity, acidity, hardness, thickness and instrumental color. On the other hand, higher contents of ash, dietary fiber, ascorbic acid, anthocyanins, total phenolic compounds and antioxidant activity are observed after eggplant flour addition, which improves the nutritional profile of the product. Thus, an added product of eggplant flour is a possible alternative to promote healthier feeding among school-age children.

For further studies, it is suggested to include other vegetable flours and their by-products in cookies and similar products. The development of flour blends is also encouraged, ones that contribute to the nutritional aspects of the food and may be an innovative option for the industry and the consumer.

## Figures and Tables

**Table 1:** Sensory scores (mean ± standard deviation) of cookies added with different eggplant flour concentrations.

Parameter	EF0	EF2.5	EF5.0	EF7.5
Appearance	6.1 ± 1.08 ^a^	5.9 ± 1.30 ^a^	5.7 ± 1.32 ^a^	5.2 ± 1.78 ^b^
AI (%)	87.8	84.4	81.2	74.7
Flavor	6.1 ± 1.23 ^a^	6.0 ± 1.27 ^a^	5.7 ± 1.41 ^a^	5.2 ± 1.87 ^b^
AI (%)	87.3	85.9	81.1	74.0
Taste	6.4 ± 1.13 ^a^	6.1 ± 1.33 ^a^	5.9 ± 1.44 ^a^	5.4 ± 1.83 ^b^
AI (%)	90.7	87.8	84.6	77.6
Texture	6.0 ± 1.18 ^a^	5.9 ± 1.28 ^a^	5.7 ± 1.31 ^a^	5.3 ± 1.69 ^b^
AI (%)	86.1	84.4	81.4	75.2
Color	6.2 ± 1.09 ^a^	6.0 ± 1.30 ^a^	5.7 ± 1.32 ^a^	5.2 ± 1.67 ^b^
AI (%)	88.2	86.0	82.0	74.0
Overall Acceptance	4.6 ± 0.69 ^a^	4.4 ± 0.86 ^a^	4.3 ± 0.84 ^a^	4.0 ± 1.26 ^b^
AI (%)	92.0	88.4	86.4	79.7
Purchase Intention	4.6 ± 0.72 ^a^	4.5 ± 0.95 ^a^	4.2 ± 0.95 ^a^	3.9 ± 1.30 ^b^

Distinct letters on the same line indicate significant difference in the Tukey’s test (*p* < 0.05); AI: Acceptability Index. The 7-point hedonic scale of attributes being 1 to 7; 5-point facial hedonic scale of overall acceptance and purchase intention being 1 to 5.

**Table 2:** Physical parameters (mean ± standard deviation) of cookie added with different eggplant flour concentrations.

Parameter	EF0	EF2.5	EF5.0	EF7.5
Diameter (cm)	3.7 ± 0.07 ^b^	3.8 ± 0.08 ^b^	4.0 ± 0.09 ^a^	4.0 ± 0.05 ^a^
Thickness (cm)	1.1 ± 0.04 ^a^	1.1 ± 0.05 ^a^	0.9 ± 0.03 ^b^	0.9 ± 0.02 ^b^
Expansion factor	3.4 ± 0.19 ^b^	3.6 ± 0.22 ^b^	4.3 ± 0.20 ^a^	4.4 ± 0.09 ^a^
Thermal factor	0.9 ± 0.00 ^b^	0.9 ± 0.00 ^b^	1.0 ± 0.03 ^a^	1.0 ± 0.00 ^a^
Hardness (N)	32.4 ± 2.54 ^d^	46.9 ± 2.73 ^c^	64.1 ± 1.96 ^b^	87.2 ± 3.02 ^a^
L*	60.5 ± 0.38 ^a^	54.1 ± 0.58 ^b^	54.0 ± 0.53 ^b^	51.6 ± 0.85 ^c^
a*	6.5 ± 0.34 ^a^	5.6 ± 0.29 ^b^	5.5 ± 0.33 ^b^	5.1 ± 0.30 ^c^
b*	24.5 ± 0.37 ^a^	20.9 ± 0.67 ^b^	20.4 ± 0.64 ^b^	18.9 ± 0.59 ^c^
Aw	0.6 ± 0.01 ^c^	0.7 ± 0.00 ^b^	0.7 ± 0.00 ^b^	0.8 ± 0.00 ^a^

Distinct letters on the same line indicate significant difference in the Tukey’s (*p* < 0.05). Aw: water activity.

**Table 3:** Chemical characteristics (mean ± standard deviation) of eggplant flour and cookies added with different eggplant flour concentrations.

Parameter	EF	EF0	EF2.5	EF5.0	EF7.5
pH	4.9 ± 0.01	6.8 ± 0.01 ^a^	6.4 ± 0.01 ^b^	6.1 ± 0.01 ^c^	5.9 ± 0.01 ^d^
TA (g 100 g^−1^)	4.1 ± 0.04	0.1 ± 0.01 ^d^	0.2 ± 0.00 ^c^	0.3 ± 0.02 ^b^	0.4 ± 0.02 ^a^
SS (°Brix)	5.0 ± 0.06	1.9 ± 0.03 ^c^	2.3 ± 0.03 ^b^	2.5 ± 0.06 ^a^	2.6 ± 0.03 ^a^
SS/TA ratio	1.2 ± 0.01	15.3 ± 0.28 ^a^	11.7 ± 0.09 ^b^	7.7 ± 0.19 ^c^	6.6 ± 0.09 ^d^

Distinct letters on the same line indicate significant difference in the Tukey’s (*p* < 0.05); TA: Titratable Acidity; SS: Soluble Solids; SS/TA ratio: Soluble Solids/Titratable Acidity ratio.

**Table 4:** Nutritional composition and antioxidant activity (mean ± standard deviation) of eggplant flour and cookies added with different eggplant flour concentrations.

Parameter	EF	EF0	EF2.5	EF5.0	EF7.5
Moisture (g 100 g^−1^)	5.5 ± 0.05	7.7 ± 0.08 ^c^	9.7 ± 0.04 ^b^	9.5 ± 0.09 ^b^	10.5 ± 0.08 ^a^
Ash (g 100 g^−1^)	7.4 ± 0.08	0.8 ± 0.01 ^d^	1.0 ± 0.01 ^c^	1.2 ± 0.01 ^b^	1.4 ± 0.02 ^a^
Protein (g 100 g^−1^)	11.4 ± 0.56	10.1 ± 0.03 ^a^	10.4 ± 0.63 ^a^	10.4 ± 0.01 ^a^	11.0 ± 0.22 ^a^
Lipid (g 100 g^−1^)	2.1 ± 0.53	19.2 ± 0.58 ^a^	19.3 ± 0.22 ^a^	19.4 ± 0.32 ^a^	19.2 ± 0.34 ^a^
Carbohydrate (g 100 g^−1^) **	39.1 ± 0.00	60.8 ± 0.00 ^a^	57.4 ± 0.00 ^ab^	56.5 ± 0.00 ^b^	54.1 ± 0.00 ^b^
Soluble fiber (g 100 g^−1^) ***	7.2 ± 0.18	0.3 ± 0.00 ^d^	0.4 ± 0.00 ^c^	0.6 ± 0.00 ^b^	0.8 ± 0.00 ^a^
Insoluble fiber (g 100 g^−1^) ***	27.3 ± 0.13	1.1 ± 0.00 ^d^	1.8 ± 0.00 ^c^	2.4 ± 0.00 ^b^	3.0 ± 0.00 ^a^
Total fiber (g 100 g^−1^) ***	34.5 ± 0.05	1.4 ± 0.00 ^d^	2.2 ± 0.00 ^c^	3.0 ± 0.00 ^b^	3.8 ± 0.00 ^a^
Energy value (kcal 100 g^−1^)	290.3 ± 0.00	458.9 ± 0.00 ^a^	449.2 ± 0.00 ^ab^	448.0 ± 0.00 ^b^	440.6 ± 0.00 ^b^
Ascorbic acid (mg 100 g^−1^)	64.0 ± 0.37	0.3 ± 0.00 ^d^	2.7 ± 0.33 ^c^	4.2 ± 0.19 ^b^	5.3 ± 0.10 ^a^
Anthocyanins (mg C3GE 100 g^−1^)	105.7 ± 0.04	2.55 ± 0.01 ^d^	24.0 ± 0.04 ^c^	26.9 ± 0.08 ^b^	29.6 ± 0.06 ^a^
TPC (mg GAE 100 g^−1^)	276.4 ± 0.08	9.6 ± 0.06 ^d^	14.8 ± 0.01 ^c^	22.7 ± 0.04 ^b^	32.2 ± 0.05 ^a^
AA (µmol Trolox equivalents 100 g^−1^)	19,117.1 ± 0.00	1786.3 ± 0.26 ^d^	1900.6 ± 0.26 ^c^	19,158 ± 0.26 ^b^	2144.3 ± 0.53 ^a^

Distinct letters on the same line indicate significant difference in the Tukey’s (*p* < 0.05); Values presented on dry basis; ** Includes dietary fiber; *** Dietary fiber. TPC: Total Phenolic Compounds; AA: Antioxidant Activity.

## Data Availability

Data is contained within the article.

## References

[1] Niño-Medina G., Urías-Orona V., Rangel M.D.M., Heredia J.B. (2017). Structure and content of phenolics in eggplant (Solanum melongena)—A review. S. Afr. J. Bot..

[2] Gurbuz N., Uluişik S., Frary A., Frary A., Doğanlar S. (2018). Health benefits and bioactive compounds of eggplant. Food Chem..

[3] Food and Agriculture Organization of the United Nations (FAO) Faostat—Production. http://www.fao.org/faostat/en/#data/QC.

[4] Instituto Brasileiro de Geografia e Estatística (IBGE) Censo Agropecuário 2017—Horticultura. https://sidra.ibge.gov.br/pesquisa/censo-agropecuario/censo-agropecuario-2017.

[5] Uthumporn U., Woo W.L., Tajul A., Fazilah A.Y. (2015). Physico-chemical and nutritional evaluation of cookies with different levels of eggplant flour substitution. CyTA J. Food.

[6] United States Department of Agriculture (USDA) FoodData Central—Eggplant, Raw. https://fdc.nal.usda.gov/fdc-app.html#/food-details/169228/nutrients.

[7] Scorsatto M., Pimentel A.D.C., Da Silva A.J.R., Sabally K., Rosa G., De Oliveira G.M.M. (2017). Assessment of Bioactive Compounds, Physicochemical Composition, and In Vitro Antioxidant Activity of Eggplant Flour. Int. J. Cardiovasc. Sci..

[8] Barros E.R.S., Azevedo D.M.S., Caldeira J.O., Sousa B.R., Bittencourt F.d.O., Vieira V.F., Duarte S.F.P., Fogaça L.C. (2019). Sensory Assessment of Cookie Type Biscuits Produced with Eggplant Flour (*Solanum Melongena*, L.). Int. J. Curr. Res..

[9] United States Per Capita Consumption of Fresh Vegetables in the United States in 2019, by Vegetable Type (in Pounds). https://www.statista.com/statistics/257345/per-capita-consumption-of-fresh-vegetables-in-the-us-by-type/.

[10] Mennella J.A., Pepino M.Y., Duke F.F., Reed D.R. (2010). Age modifies the genotype-phenotype relationship for the bitter receptor TAS2R38. BMC Genet..

[11] Cain J.P., Silva V.D.C., Franco B.C., Da Luz L.A.P., Dos Santos E.F., Novello D. (2020). Oficinas de culinária melhoram a aceitabilidade de alimentos entre crianças de idade escolar. Res. Soc. Dev..

[12] Rossi M.C., Bassett M.N., Sammán N.C. (2018). Dietary nutritional profile and phenolic compounds consumption in school children of highlands of Argentine Northwest. Food Chem..

[13] World Health Organization Healthy Diet. https://www.who.int/who-documents-detail-redirect/healthy-diet-factsheet394.

[14] Teixeira F., De Lima K.A., Silva V.D.C., Franco B.C., Dos Santos E.F., Novello D. (2018). Farinha da casca de berinjela em pão: Análise físico-química e sensorial entre crianças. Cienc. Saude.

[15] da Rosa P.A., Rodrigues B.M., dos Santos N.M., Candido C.J., dos Santos E.F., Novello D. (2016). Elaboração de esfihas de frango adicionadas de farinha de casca de berinjela: Análise físico-química e sensorial. Revista Uniabeu.

[16] Rodriguez-Jimenez J.R., Amaya-Guerra C.A., Baez-Gonzalez J.G., Aguilera-Gonzalez C., Urias-Orona V., Nino-Medina G. (2018). Physicochemical, Functional, and Nutraceutical Properties of Eggplant Flours Obtained by Different Drying Methods. Molecules.

[17] Mbondo N.N., Owino W.O., Ambuko J., Sila D.N. (2018). Effect of drying methods on the retention of bioactive compounds in African eggplant. Food Sci. Nutr..

[18] Associação Brasileira das Indústrias de Biscoitos, Massas Alimentícias e Pães & Bolos Industrializados (ABIMAPI) Estatísticas. https://www.abimapi.com.br/estatisticas-biscoitos.php.

[19] Martins A.P.B., Levy R.B., Claro R.M., Moubarac J.C., Monteiro C.A., Martins A.P.B., Levy R.B., Claro R.M., Moubarac J.C., Monteiro C.A. (2013). Increased Contribution of Ultra-Processed Food Products in the Brazilian Diet (1987–2009). Revista De Saude Publica.

[20] Chavan R.S., Sandeep K., Basu S., Bhatt S. (2016). Biscuits, Cookies, and Crackers: Chemistry and Manufacture. Encyclopedia of Food and Health.

[21] Aboshora W., Yu J., Omar K.A., Li Y., Hassanin H.A.M., Navicha W.B., Zhang L. (2019). Preparation of Doum fruit (Hyphaene thebaica) dietary fiber supplemented biscuits: Influence on dough characteristics, biscuits quality, nutritional profile and antioxidant properties. J. Food Sci. Technol..

[22] United States (2017). 2015–2020 Dietary Guidelines for Americans.

[23] Brazil (2015). Boas Práticas de Manipulação de Alimentos.

[24] Kroll B.J. (1990). Evaluating Rating Scales for Sensory Testing with Children. Food Qual. Prefer..

[25] Guimarães R.R., Vendramini A.L.D.A., dos Santos A.C., Leite S.G.F., Miguel M.A.L. (2013). Development of probiotic beads similar to fish eggs. J. Funct. Foods.

[26] American Association of Cereal Chemists (AACC) (2000). Approved Methods.

[27] Konica Minolta (2007). Precise Color Communication: Color Control from Perception to Instrumentation.

[28] Association of Official Analytical Chemists (AOAC) (2016). Official Methods of Analysis of AOAC International.

[29] Bligh E.G., Dyer W.J. (1959). A rapid method of total lipid extraction and purification. Can. J. Biochem. Physiol..

[30] Atwater W.O., Woods C.D. (1896). The Chemical Composition of American Food Materials.

[31] Institute of Medicine (2005). Dietary Reference Intakes for Energy, Carbohydrate, Fiber, Fat, Fatty Acids, Cholesterol, Protein, and Amino Acids.

[32] Benassi M.T., Antunes A.J. (1988). A Comparison of Metaphosphoric and Oxalic Acids as Extractants Solutions for the Determination of Vitamin C in Selected Vegetables. Arch. Biol. Technol..

[33] Giusti M.M., Wrolstad R.E. (2001). Characterization and Measurement of Anthocyanins by UV-Visible Spectroscopy. Curr. Protoc. Food Anal. Chem..

[34] Woisky R.G., Salatino A. (1998). Analysis of propolis: Some parameters and procedures for chemical quality control. J. Apic. Res..

[35] Re R., Pellegrini N., Proteggente A., Pannala A., Yang M., Rice-Evans C. (1999). Antioxidant activity applying an improved ABTS radical cation decolorization assay. Free Radic. Biol. Med..

[36] R CORE TEAM (2019). R: A Language and Environment for Statistical Computing.

[37] Hanson K.L., Garner J., Connor L.M., Pitts S.B.J., McGuirt J., Harris R., Kolodinsky J., Wang W., Sitaker M., Ammerman A. (2019). Fruit and Vegetable Preferences and Practices May Hinder Participation in Community-Supported Agriculture Among Low-Income Rural Families. J. Nutr. Educ. Behav..

[38] Drewnowski A., Gomez-Carneros C. (2000). Bitter taste, phytonutrients, and the consumer: A review. Am. J. Clin. Nutr..

[39] Vecchio R., Cavallo C., Cicia G., Del Giudice T. (2019). Are (All) Consumers Averse to Bitter Taste?. Nutrients.

[40] Brasil D.L., Belo T.A.R., Zambelli R.A., Pontes D.F., Silva M.L. (2014). Desenvolvimento de Pães Tipo Forma Adicionado de Farinha de Berinjela. Anais do XX Congresso Brasileiro de Engenharia Química.

[41] Chung L.M.Y., Fong S.S.M., Chung J.W.Y. (2016). Identification on Sensory Attributes and Children’s Ratings of Fruits and Vegetables with and without Appearance Modification: A Pilot Study. J. Child Adolesc. Behav..

[42] Mauro A.K., da Silva V.L.M., Freitas M.C.J. (2010). Caracterização física, química e sensorial de cookies confeccionados com farinha de talo de couve (FTC) e farinha de talo de espinafre (FTE) ricas em fibra alimentar. Ciênc. Technol. Aliment..

[43] Noda Y., Kneyuki T., Igarashi K., Mori A., Packer L. (2000). Antioxidant activity of nasunin, an anthocyanin in eggplant peels. Toxicology.

[44] Valerga L., Quintero-Ruiz N.A., Concellón A., Puppo M.C. (2020). Technological and Nutritional Characterization of Wheat Breads Added with Eggplant Flour: Dependence on the Level of Flour and the Size of Fruit. J. Food Sci. Technol..

[45] Sociedade Brasileira de Pediatria (SBP) (2018). Manual de Alimentação: Orientações Para Alimentação Do Lactente Ao Adolescente Na Escola, Na Gestante, Na Prevenção de Doenças e Segurança Alimentar.

[46] Saueressig A.L.C., Kaminski T.A., Escobar T.D. (2016). Inclusion of Dietary Fiber in Gluten-Free Breads. Braz. J. Food Technol..

[47] Perez P.M.P., Germani R. (2007). Elaboração de biscoitos tipo salgado, com alto teor de fibra alimentar, utilizando farinha de berinjela (*Solanum melongena*, L.). Ciênc. Tecnol. Aliment..

[48] Kurek M.A., Wyrwisz J., Piwińska M., Wierzbicka A. (2015). Influence of the wheat flour extraction degree in the quality of bread made with high proportions of β-glucan. Food Sci. Technol..

[49] Koponen J.M., Happonen A.M., Mattila P.H., Törrönen A.R. (2007). Contents of Anthocyanins and Ellagitannins in Selected Foods Consumed in Finland. J. Agric. Food Chem..

[50] Clerici M.T.P.S., de Oliveira M.E., Nabeshima E.H. (2013). Qualidade física, química e sensorial de biscoitos tipo cookies elaborados com a substituição parcial da farinha de trigo por farinha desengordurada de gergelim. Braz. J. Food Technol..

[51] Simmonds M.S.J., Preedy V.R. (2016). Nutritional Composition of Fruit Cultivars.

[52] Perez P.M.P., Germani R. (2004). Farinha Mista de Trigo e Berinjela: Características Físicas e Químicas. ALICE.

[53] de Souza V.R., Pereira P.A.P., Queiroz F., Borges S.V., Deus Souza Carneiro J. (2012). Determination of bioactive compounds, antioxidant activity and chemical composition of Cerrado Brazilian fruits. Food Chem..

[54] Brazil (2005). Resolução RDC No 263, de 22 de Setembro de 2005. Regulamento Técnico Para Produtos de Cereais, Amidos, Farinhas e Farelos.

[55] Mexico. Norma Oficial Mexicana, NOM-247-SSA1-2008, Productos y Servicios. Cereales y sus Productos. Cereales, Harinas de Cereales, Sémolas o Semolinas. Alimentos a Base de: Cereales, Semillas Comestibles, de Harinas, Sémolas o Semolinas o sus Mezclas. Productos de Panificación. Disposiciones y Especificaciones Sanitarias y Nutrimentales. Métodos de Prueba. http://depa.fquim.unam.mx/amyd/archivero/NOMcereales_12434.pdf.

[56] United States Department of Agriculture (USDA) (2017). USDA Commodity Requirements—WF16 Wheat Flour Products for Use in Domestic Programs.

[57] Rosell C.M., Rojas J.A., Benedito de Barber C. (2001). Influence of hydrocolloids on dough rheology and bread quality. Food Hydrocoll..

[58] Merkiel S., Chalcarz W. (2014). Dietary intake in 6-year-old children from southern Poland: Part 2—vitamin and mineral intakes. BMC Pediatr..

[59] Bundy D.A.P., De Silva N., Horton S., Jamison D.T., Patton G.C. (2017). Child and Adolescent Health and Development.

[60] United States Department of Agriculture (USDA) FoodData Central—Wheat Flour, White, Bread, Enriched. https://fdc.nal.usda.gov/fdc-app.html#/food-details/168896/nutrients.

[61] Brazil (2012). Resolução RDC No 54, de 12 de Novembro de 2012. Regulamento Técnico Mercosul Sobre Informação Nutricional Complementar (Declarações de Propriedades Nutricionais).

[62] Frond A.D., Iuhas C.I., Stirbu I., Leopold L., Socaci S., Andreea S., Ayvaz H., Andreea S., Mihai S., Diaconeasa Z. (2019). Phytochemical Characterization of Five Edible Purple-Reddish Vegetables: Anthocyanins, Flavonoids, and Phenolic Acid Derivatives. Molecules.

